# Multiple areas of the cerebral cortex influence the stomach

**DOI:** 10.1073/pnas.2002737117

**Published:** 2020-05-20

**Authors:** David J. Levinthal, Peter L. Strick

**Affiliations:** ^a^University of Pittsburgh Brain Institute, University of Pittsburgh School of Medicine, Pittsburgh, PA 15261;; ^b^Neurogastroenterology & Motility Center, Division of Gastroenterology, Hepatology, and Nutrition, Department of Medicine, University of Pittsburgh School of Medicine, Pittsburgh, PA 15261;; ^c^Systems Neuroscience Center, University of Pittsburgh School of Medicine, Pittsburgh, PA 15261;; ^d^Department of Neurobiology, University of Pittsburgh School of Medicine, Pittsburgh, PA 15261

**Keywords:** interoception, rabies, autonomic, parasympathetic, sympathetic

## Abstract

The central nervous system both influences and is influenced by the gastrointestinal system. Most research on this gut–brain connection has focused on how ascending signals from the gut and its microbiome alter brain function. Less attention has focused on how descending signals from the central nervous system alter gut function. Here, we used retrograde transneuronal transport of rabies virus to identify the cortical areas that most directly influence parasympathetic and sympathetic control of the rat stomach. Cortical neurons that influence parasympathetic output to the stomach originated from the rostral insula and portions of medial prefrontal cortex, regions that are associated with interoception and emotional control. In contrast, cortical neurons that influence sympathetic output to the stomach originated overwhelmingly from the primary motor cortex, primary somatosensory cortex, and secondary motor cortex, regions that are linked to skeletomotor control and action. Clearly, the two limbs of autonomic control over the stomach are influenced by distinct cortical networks.

## Significance

Despite the longstanding appreciation that how we move, think, and feel has an impact on stomach function, the areas of the cerebral cortex that are the origin of these influences are largely unknown. Here we identify the cortical areas that influence the rat stomach. Output neurons in the rostral insula are the major cortical source of influence over parasympathetic control of the stomach, whereas output neurons in sensorimotor areas of the cortex are the major source of influence over sympathetic control. Thus, cortical areas involved in action, interoception, and emotion differentially influence stomach function.

The central nervous system both influences and is influenced by the gastrointestinal system. Most research on this gut–brain connection has focused on the ascending pathways that link signals from the gut and its microbiome to alterations in brain function (1, 2). Less attention has been devoted to the descending pathways that link brain operations to the function of the gut. Yet it has long been known that the central nervous system uses environmental signals and predictions from past experience to generate anticipatory responses that promote efficient digestion (3, 4).

Central control over stomach function is mediated by the parasympathetic and sympathetic limbs of the autonomic nervous system. In general, parasympathetic output to the stomach tends to increase secretions and enhance the patterns of smooth muscle contractility that are required for processing a meal (5–7). In contrast, sympathetic output to the stomach tends to decrease secretions and inhibit these patterns of smooth muscle contractility (8–10). Both sets of outputs alter the microenvironment of the stomach, and thus its microbiome, by controlling the exposure of ingested bacteria to acid, proteolytic enzymes, mucin, and immune factors (11, 12).

Given the importance of the central control over stomach function, it is surprising how little is known about the cortical origins of the descending commands that mediate these effects. Here, we used retrograde transneuronal transport of rabies virus (RV) to reveal the chain of interconnected neurons that links the cerebral cortex to parasympathetic and sympathetic control of the rat stomach. This species is of particular interest because of its rapid development of stress-induced stomach ulcers (13, 14). We demonstrate that separate cortical networks influence parasympathetic and sympathetic control of the stomach.

## Results

**Fig. 1.**
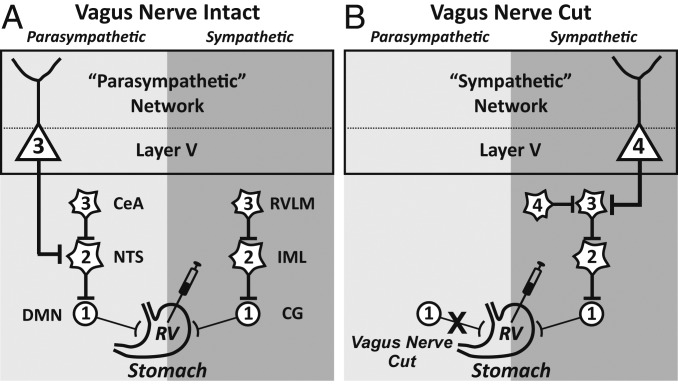
Schematic of the experimental paradigm. The patterns of retrograde transneuronal transport seen after injections of RV into the anterior wall of the stomach. Each stage of transport is numbered. (*A*) The survival time was adjusted to restrict transport to third-order neurons, such as output neurons in cortical Layer V that influence parasympathetic function. (*B*) The anterior and posterior vagus nerves were sectioned and the survival time was adjusted to restrict transport to fourth-order neurons, such as output neurons in cortical Layer V that influence sympathetic function. CeA, central nucleus of the amygdala; CG, celiac ganglion; DMN, dorsal motor nucleus of the vagus; IML, intermediolateral nucleus of the spinal cord; RVLM, rostral ventrolateral medulla.

We injected RV into the anterior wall of the rat stomach and used retrograde transneuronal transport of the virus to label the cortical neurons that most directly influence this organ. We confined our analysis to cases in which cortical neurons infected with RV were restricted to Layer V, the origin of subcortical outputs from the cerebral cortex. To isolate cortical labeling to parasympathetic or sympathetic circuits, we carefully adjusted survival times and employed bilateral subdiaphragmatic vagotomy in some animals (Fig. 1).

### Parasympathetic Network.

**Fig. 2.**
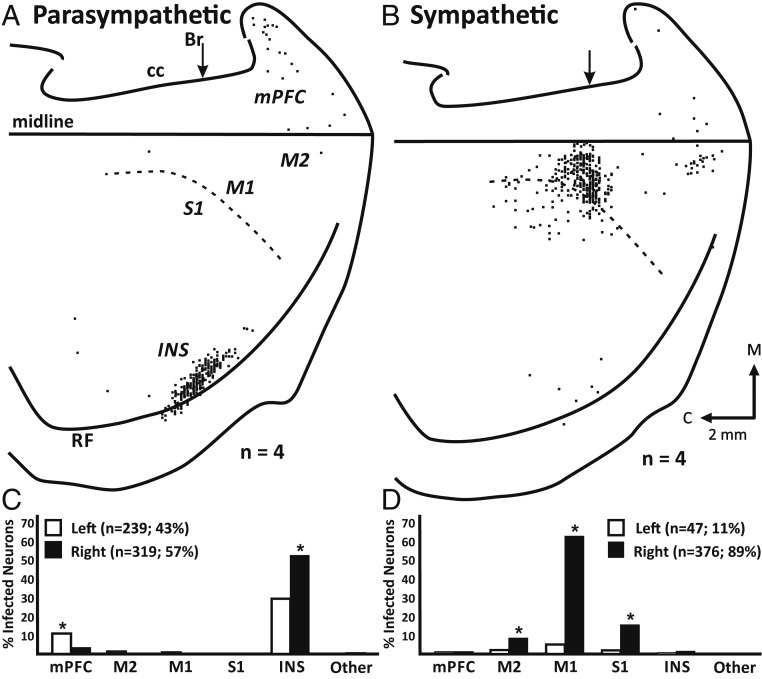
Cortical networks that influence parasympathetic or sympathetic control of the rat stomach. (*A*) Parasympathetic network: Flattened cortical map of third-order neurons labeled in Layer V of the right hemisphere after transport of RV from the stomach. (*B*) Sympathetic network: Flattened cortical map of fourth-order neurons labeled in Layer V of the right hemisphere after transport of RV from the stomach of animals in which the vagus nerves were sectioned. In both *A* and *B*, the results from 4 animals are overlapped in these maps. The medial wall of the hemisphere is reflected upward and joined to the lateral surface at the midline. The dashed lines indicate the border between agranular (M1) and granular (S1) cortex in the region of the forelimb, trunk, and hindlimb representations. Each small square represents a single labeled neuron. Br, bregma; C, caudal; CC, corpus callosum; INS, insula; M, medial; midline, midline of the hemisphere; mPFC, medial prefrontal cortex; M1, primary motor cortex; M2, secondary motor cortex; RF, rhinal fissure; S1, primary somatosensory cortex. (*C*) Parasympathetic network: distribution of third-order neurons in various cortical areas after transport of RV from the stomach. (*D*) Sympathetic network: distribution of fourth-order neurons in various cortical areas after transport of RV from the stomach in animals with sectioned vagus nerves. In both *C* and *D*, filled bars, right hemisphere; unfilled bars, left hemisphere.

**Fig. 3.**
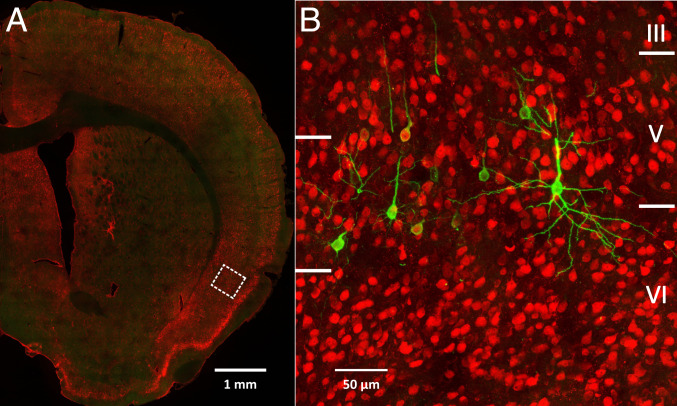
Infected third-order neurons in Layer V of the insular cortex. (*A*) Low magnification view of a coronal section through the right hemisphere. The dashed box indicates the location of the field enlarged in *B*. (*B*) Third-order neurons in Layer V of insular cortex that were labeled by RV transport from the stomach. NeuN-stained neurons are red, and RV-infected neurons are green.

Transport of RV in parasympathetic circuits labeled first-order neurons in the dorsal motor nucleus of the vagus, second-order neurons in the nucleus of the solitary tract, and third-order neurons in Layer V of the cerebral cortex (Figs. 1*A*, 2*A*, and 3). Thus, a network of three interconnected neurons is sufficient to allow the output of the cerebral cortex to influence parasympathetic control of the stomach.

The overwhelming majority of the cortical neurons that influence parasympathetic control of the stomach were located in two cortical regions: the insula (>81%) and portions of medial prefrontal cortex (infralimbic and prelimbic; 13%; Fig. 2 *A* and *C*). Several other cortical areas also contained a few isolated labeled neurons, but none of these areas contained more than 2% of the total sample of labeled neurons (Fig. 2*C*). Overall, the cortical neurons that influence parasympathetic control of the stomach were slightly more numerous in the right hemisphere (57%) than in the left hemisphere (43%; Fig. 2*C*). However, nearly twice as many insular neurons were labeled in the right hemisphere (52.0%) as in the left hemisphere (29.2%). In contrast, more than three times as many prefrontal neurons were labeled in the left hemisphere (10.4%) as in the right hemisphere (3.0%; Fig. 2*C*).

### Sympathetic Network.

Before injecting RV into the stomach, we cut the left and right vagus nerves below the diaphragm (Fig. 1*B*). This enabled us to isolate transneuronal transport of RV to sympathetic networks. Transneuronal transport of RV after vagal nerve section labeled first-order neurons in the celiac ganglia (10), second-order neurons in the intermediolateral column of the spinal cord, third-order neurons in the rostral ventrolateral medulla of the brainstem, and fourth-order neurons in Layer V of the cerebral cortex (Figs. 1*B* and 2*B*). Thus, a network of 4 interconnected neurons is sufficient to allow the output of the cerebral cortex to influence sympathetic control of the stomach.

The overwhelming majority of the cortical neurons that influence sympathetic control of the stomach were located in three cortical areas: primary motor cortex (M1; 62.2%), primary somatosensory cortex (S1; 15.4%), and secondary motor cortex (M2; 8.3%; Fig. 2 *B* and *D*). A few, isolated neurons also were located in several other cortical areas, but none of these cortical areas contained more than 2% of the total sample of labeled neurons (Fig. 2*D*). Overall, the cortical neurons that influence sympathetic control of the stomach were located in the right hemisphere (89%; Fig. 2*D*).

**Fig. 4.**
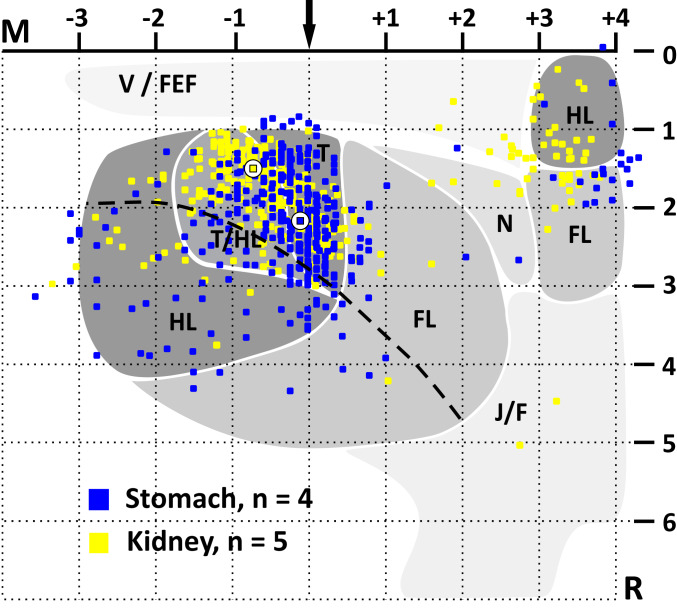
Viscerotopic organization of Layer V output neurons in M1 and M2 that influence sympathetic control of the stomach or kidney. Each colored square represents a single neuron labeled by retrograde transneuronal transport of RV from the stomach (blue) or kidney (yellow). The kidney data were taken from Levinthal and Strick (15). The data are overlaid on maps of motor representation in M1 and M2 (for details, see ref. 15). Although the two groups of labeled neurons show considerable overlap, the peak density of Layer V neurons that influence the stomach (circle with blue square) is slightly rostral and lateral to the peak density of Layer V neurons that influence the kidney (circle with yellow square). FL, forelimb; HL, hindlimb; J/F, jaw/face; M, medial; N, neck; R, rostral; T, trunk; T/HL, trunk/hindlimb overlap zone; V/FEF, vibrissae/frontal eye fields. The maps are aligned on bregma (arrow) and Layer V at the midline (the horizontal dashed line at 1 mm). The curved dashed line indicates the border between agranular (M1) and granular (S1) cortex in the fore- and hindlimb areas.

The presence of a fairly well-defined body map in M1/S1 allowed us to determine which body representations contained output neurons that influence sympathetic control of the stomach. In the right hemisphere, these output neurons were largely confined (>80%) to the trunk and trunk/hindlimb representations (Fig. 4, blue squares). The small number of neurons seen in the left hemisphere were similarly located, although this issue was not quantitatively analyzed.

We previously identified the cortical areas that influence sympathetic control of the kidney (15). We added these data to the M1/S1 body map (Fig. 4, yellow squares) to compare the distributions of the two sets of output neurons. This comparison demonstrated that the two populations overlap considerably in M1/S1. Even so, the center of mass of the output neurons in M1/S1 that influence the stomach (Fig. 4, circle with blue square) is shifted 0.6 mm rostral and 0.7 mm lateral to the center of mass of the output neurons in M1/S1 that influence the kidney (Fig. 4, circle with yellow square). The stomach and kidney are innervated by largely different segments of the spinal cord (stomach: T6-T10, ref. 16; kidney: T10-T12, ref. 17). Thus, our results suggest that M1/S1 contains a viscerotopic map of stomach and kidney representation. This map is embedded within the classic somatotopic organization of M1/S1.

## Discussion

The two components of the autonomic nervous system, parasympathetic and sympathetic, have commonly been characterized in very distinct ways: “rest and digest” (involving internal, vegetative processes) and “fight or flight” (involving action). Although these characterizations are an oversimplification, and autonomic regulation is more nuanced and predictive (18, 19), these terms reflect the different effects evoked by the two systems. Our results clearly demonstrate that distinct cortical areas are the source of descending control over each component of autonomic output to the stomach. One cortical network originates from areas linked to interoception and emotion, and the other cortical network originates from areas involved in action.

**Fig. 5.**
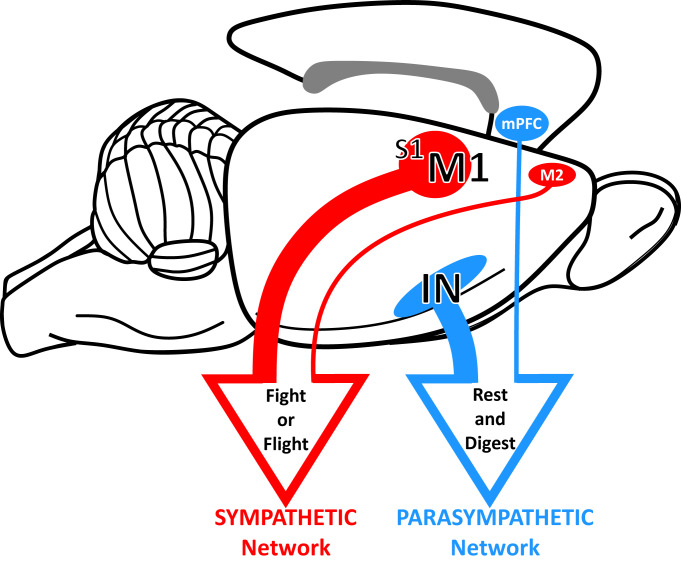
Cortical networks for autonomic control of the stomach. Distinct cortical networks influence parasympathetic and sympathetic output to the stomach.

The rostral insula is the major cortical source of descending control over parasympathetic output to the stomach (Figs. 2*A* and 5). In fact, stimulation of this region of the insula is known to evoke changes in gastric motor function that are consistent with increased parasympathetic drive to the stomach (20). An additional smaller source of descending control originates from selected regions of the rat’s medial prefrontal cortex (Figs. 2*A* and 5). This result is consistent with the finding that alterations in gastric function can be evoked by microstimulation in this region (21).

**Fig. 6.**
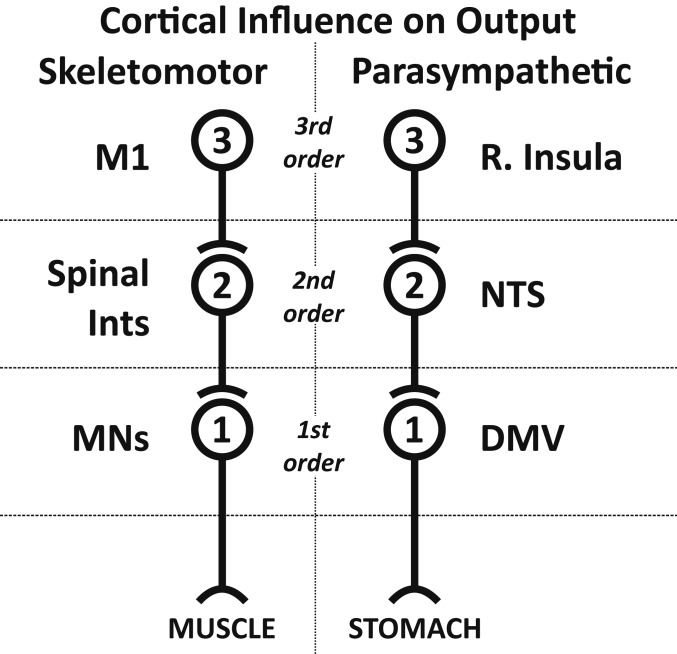
Cortical control of skeletomotor and parasympathetic output. A similar network architecture characterizes the two systems.

Our results indicate that the rostral insula is linked to the stomach by a series of three synaptically connected neurons (Fig. 6, *Right*). This network architecture is comparable to the series of three synaptically connected neurons that link the output from the primary motor cortex to skeletal muscles in the rat (Fig. 6, *Left*). Thus, the rostral insula may serve as a parasympathetic motor cortex with a command structure and function that is comparable to the control of skeletal muscle by the motor cortex.

The rostral insula gathers visceral afferent information, including signals from the stomach (22, 23), and has been viewed as a visceral sensory cortex that is critical for interoception (24). A comparison of our results with prior data (23) suggests that the sensory and motor functions of the rostral insula overlap. If so, this arrangement creates a loop (stomach → cortex → stomach) and implies that so-called gut feelings triggered by afferent signals from the stomach can be conditioned by descending commands from the same cortical area. This anatomical arrangement fits with the perspective that interoception and emotion are constructed on the basis of a complex interplay between afferent signals from organs and central encoding of past experiences and context (25). Our results emphasize the potential importance of descending commands from a variety of cortical areas in this construction process. Our findings also highlight the potential for central commands to influence the afferent signals from the stomach through their control over autonomic output.

In the rat, the descending control over sympathetic output to the stomach is embedded in M1/S1 and M2 (Figs. 2*B* and 5). This is also the case for the descending control over sympathetic output to the rodent kidney and adrenal medulla (15, 26). A similar situation exists in the monkey, where the cortical motor areas in the frontal lobe are a major source of the descending control over the adrenal medulla (27). In general, these motor areas are involved in a broad range of motor activities including the generation of specific parameters of movement, as well as the preparation to move and the selection of actions (28, 29). The colocalization of skeletomotor and sympathetic function within the same cortical areas may represent a specific mechanism to facilitate the coordination of sympathetic and skeletomotor actions in a wide range of behavioral circumstances.

The viscerotopic shifts in the location of cortical neurons that influence sympathetic output (Fig. 4) are similar to the somatotopic shifts in the location of cortical neurons that influence skeletomotor output (29). Both appear to reflect the spinal segmental organization of the two systems. Somatotopic shifts are thought to provide a substrate that enables differential control of specific muscles. Perhaps the viscerotopic organization we have observed provides a similar substrate for differential control of specific organs.

It is also noteworthy that the cortical distributions of the output neurons innervating the stomach and kidney display considerable overlap. This arrangement is similar to the overlap observed between the cortical distributions of output neurons innervating synergistic muscles. In both cases, the partially shifted overlap may be the substrate for variable, but integrated, control of the different output systems.

There has been a growing awareness of the importance of the gut–brain axis to human health. However, the discussion of this issue has largely focused on how the gut microbiome influences the function of other organ systems (1, 2, 30–32). Our results suggest that the gut–brain axis should also be viewed from another perspective; that is, how signals from the brain influence the gut microbiome. As we noted here, the balance of activation in the two autonomic drives to the stomach can tune the gastric microenvironment. Stomach content has a strong influence on the composition of the microbiome that is passed on to more distal regions of the gastrointestinal tract (11, 12). Thus, it is possible that transient or sustained cortical activation can have a profound impact on the composition of the gut microbiome.

Ulcer formation provides one concrete example of the interaction between central signals and the stomach’s microbiome. For more than a century, every increase in unemployment and its associated stress was accompanied by an increase in death rates from stomach ulcers (33). We now know that a proximal cause of ulcer formation is often infection by *Helicobacter pylori* (34). However, the growth conditions for this bacterium can be influenced by parasympathetic command signals communicated by the vagus nerve, and selective gastric vagotomy was a common successful intervention (35). Our current finding of direct cerebral control over parasympathetic output to the stomach elucidates a mechanism for a significant psychosomatic contribution to this problematic disease.

Finally, the so-called functional gastrointestinal disorders, especially those that are severe, are often refractory to conventional treatments (36). There is increasing evidence that nonpharmacologic therapies can have positive and long-lasting therapeutic benefits (37–41). Our results provide cortical targets for brain-based therapies for functional gastrointestinal disorders. This could involve altering stomach function and/or the microbiome through the engagement of specific cortical areas, using noninvasive transcranial stimulation alone or combined with cognitive-, behavioral-, and movement-based therapies. In any event, our results provide a concrete neural basis for the concept that specific areas of the cerebral cortex differentially control stomach function.

## Materials and Methods

Our observations are based on male Sprague–Dawley rats (weight range, 250 to 275 g) that received RV injections into the anterior stomach wall. We used the N2c strain of RV (CVS-N2c; 5 × 10^8^ pfu/mL; M. Schnell, Thomas Jefferson University). Most of the technical procedures, as well as those for handling virus and virus-infected animals, have been described elsewhere (15, 42) and will be only briefly reviewed here. These procedures were approved by the relevant Institutional Animal Care and Biosafety Committees. Biosafety practices conformed to Biosafety Level 2+ regulations outlined in Biosafety in Microbiological and Biomedical Laboratories. It is important to note that none of the animals infected with RV during these experiments displayed symptoms specifically associated with RV.

### Stomach Injections.

All surgeries were conducted under sterile conditions. We induced general anesthesia, using injections of ketamine (75 mg/kg intramuscularly) and xylazine (4 mg/kg intramuscularly), and all animals received perioperative analgesia with buprenorphine (0.1 mg/kg subcutaneously). The stomach was accessed via an anterior midline incision. The gastrohepatic ligament was cut to allow the liver to be reflected superiorly and expose the full extent of the anterior wall of the stomach. We placed eight injections of RV (∼4.0 μL each, using a Hamilton microsyringe fitted with a 30-gauge needle) into a region of the distal fundus and proximal body of the stomach. Following the injections, we sutured the wound in layers and then returned each animal to an isolation cage specifically designed for housing virus-infected rats.

### Vagal Nerve Sections.

We placed animals on a liquid diet (DietGel, ClearH2O) for 48 h before surgery. To cut the vagus nerve, we retracted the liver to reveal the subdiaphragmatic portion of the esophagus. The anterior vagal trunk was identified and cut just proximal to the gastroesophageal junction. We cut the posterior vagal trunk at a more proximal level. Immediately following the nerve sections, we injected the stomach with RV as described earlier. Because vagal denervation of the stomach leads to gastric stasis and potentially impairs tolerance for solid foods, we provided these animals with a diet composed of a diluted nutrient drink (Ensure, Abbott) and DietGel for the entirety of the survival period.

### Survival Period.

We varied survival times to restrict labeling to Layer V neurons in the cerebral cortex. To examine parasympathetic circuits, the survival time was set to label third-order neurons (*n* = 4 animals, mean survival time, 89.5 h); to examine sympathetic circuits, the survival time was set to label fourth-order neurons (*n* = 4 animals, mean survival time, ∼114.5 h) and the vagus nerve was sectioned (Fig. 1). A control group of animals underwent vagal nerve section before the RV injection (*n* = 3 animals; mean survival time, 100 h). This control group demonstrated infection of third-order neurons in the spinal cord and brainstem, but no labeling in the cerebral cortex. At the end of the survival period, each animal was anesthetized and then perfused with blood washout (phosphate buffer) and fixative (10% buffered formalin and then 10% buffered formalin containing 10% glycerol) to preserve the nervous system (15). Following perfusion, the brain and spinal cord tissue were removed and stored at 4 °C in a solution of phospho-Tris-azide buffer and 20% glycerol.

### Histological Procedures.

We used standard immunohistochemical methods to section and react the tissue from these experiments (15). In brief, we cut serial frozen coronal sections (50 μm) of a brain block including the entire cerebral cortex, cerebellum, and brainstem. We also cut serial frozen sections (50 μm) of a spinal cord block containing the fifth through the tenth thoracic segments. To label neurons infected by RV, we used the avidin-biotin peroxidase technique (Vectastain, Vector Laboratories) on freely floating tissue sections and monoclonal antibody M957 as the primary antibody (diluted 1:300; supplied by A. Wandeler) (43). For select sections, we used mouse monoclonal antibody 31G10 as the primary antibody to detect neurons infected by rabies virus (diluted 1:1,000; supplied by M. Schnell) and rabbit anti-NeuN (diluted 1:1,000, Sigma) as the primary antibody to detect all neuronal cell bodies. Goat anti-mouse immunoglobulin G (IgG; Alexa488, diluted 1:500; Invitrogen) and goat anti-rabbit IgG (Alexa647, diluted 1:500; Invitrogen) were used as the secondary antibodies. These sections were mounted using Slowfade Gold anti-fade medium (Thermo Fisher Scientific). Every 10th section of the brain and every 20th section of the spinal cord was stained with cresyl violet for cytoarchitectonic analysis.

### Analytic Procedures.

Our analytic procedures have been described in detail previously (15). Briefly, we examined reacted sections under the microscope, using brightfield and/or darkfield polarized light illumination. The fluorescent images were captured using a prototype confocal laser scanning system (based on a Leica Application Suite Advanced Fluorescence SP5; Leica Microsystems) that was equipped with a glycerol/oil immersion objective (HC PL APO 40×, 0.75), a tandem scanning system (Resonance Scanner), spectral detectors with hybrid technology (GaAsP photocathode), and mosaic scanning software (Matrix Screener [beta version]), provided by Frank Sieckmann, Leica Microsystems. Mosaic image stacks of volumes up to 0.802 × 0.802 × 0.05 mm were acquired at a resolution of 0.15685 × 0.15685 × 0.5 μm per voxel (2.3 × digital zoom, 8 × line average, 8-kHz scanning speed, 5 × 5 fields of view for each brain section). The spectral detector settings for NeuN-Alexafluor 647 detection were 650- to 785-nm wavelength excited with a 633-nm laser, and for Rabies-Alexafluor 488, the detection settings were 495- to 550-nm wavelength excited with a 488-nm laser. Our procedures for charting the location of labeled neurons and creating flattened maps of labeled neurons in the cerebral cortex have been described in detail (44). We based the nomenclature and boundaries for cortical areas on a standard atlas of the rat brain (45).

### Statistical Analysis.

We used χ^2^ tests to compare the proportions of neurons in various cortical regions that were linked to either the parasympathetic or sympathetic regulation of the rat stomach. All *P* values were adjusted for multiple comparisons using the Bonferroni method. *P* values <0.05 were taken as statistically significant.

### Data Availability.

Data are available from the corresponding author upon request.
